# Hotel Dieu

**Published:** 1829-07

**Authors:** 


### HOTEL DIEU.

*Syphilis. Paralysis of the Facial Nerve. Cure.* (Hôtel Dieu, Saint-Jean's Ward, No. 12.)

A girl, æt. sixteen, of a healthy constitution, contracted gonorrhœa in November 1828, for which no treatment was adopted. Six weeks after, she arrived in Paris: at this time there was a tumor upon the left frontal protuberance. The day after her arrival, during the night, without any previous pain or any accidental cause, she experienced a numbness of the left cheek; the whole of the face on this side was stiff and insensible, and in the morning she perceived that her mouth was strongly drawn towards the right side. There was also a slight stiffness of the tongue, and her speech was embarrassed. No other symptom existed. Upon the first attack, she had been bled twice in the day from the arm, and on the succeeding day leeches were applied to the anus. From this treatment no benefit was obtained, and two days after the patient was admitted into the Hôtel Dieu.

The gonorrhœal discharge still existed, and the exostosis of the os frontis was very apparent. Neither the head nor stomach was affected. The motions of the tongue were free and natural; the difficulty of speaking evidently resulted from the immobility of the cheek and lips. An emetic was given on the two succeeding days, and on the third she was bled from the arm, but no alteration occurred. The antisyphilitic treatment of M. Dupuytren was now commenced, consisting of pills of an eighth of a grain of the deuto-chlorate of mercury, half a grain of opium, and two grains of extract of guaiacum. Three of these pills were taken each day, together with decoction of sarsaparilla and sudorific syrup.

Eight days after the appearance of the paralytic affection on the left side, the same symptom suddenly manifested itself on the right, and the face of the patient, when she awoke, was no longer drawn to one side. There was now a complete relaxation and a perfect immobility of all the features. The eyelids could be only half closed, and the tears flowed over the cheeks. The lips remained open, and were agitated like two curtains by the expired air. The tongue was not affected. The power of motion had alone suffered, for the skin and the mucous membranes retained their natural sensibility. The patient did not suffer, and her naturally very expressive countenance had now a serious cast, which oddly contrasted with her cheerful disposition of mind. She sometimes laughed heartily, but she appeared as if she were laughing behind a mask, and, from a knowledge of this fact, she was much annoyed.

The treatment was regularly continued, and a blister was applied on the left cheek near the ear, and several others successively on the same part of the right side of the face. A large seton was placed in the neck, which produced considerable pain; suppuration was not established from it for a month, until when its benefit was not apparent.

At the expiration of two months, the mobility of the cheeks was gradually recovered. The patient no longer slept with her mouth open. The power of closing the eyelids was by degrees restored, and the tears no longer flowed over the cheeks. It is to be observed, that none of the senses had ever been affected. Both taste and smell had remained perfect; neither had the sensibility of the skin been diminished or altered. The general health had not been disturbed; the appetite was good throughout the attack. At the commencement of the disease, however, the patient disliked to eat, because the food, on account of the immobility of the cheeks, collected in the mouth, and she had not the power of forming it into a mass, or of swallowing it. She latterly became accustomed to this state, and her tongue, fingers, and various instruments, were made to perform the duty of the buccinator and labial muscles. The muscles of the face very gradually regained the power of assisting in respiration, or of depicting any mental emotion. The patient had been observed to sneeze without presenting that peculiar expression of the face which naturally accompanies the act. When she gaped, the jaw dropped, but neither the lips nor the countenance indicated, in the slightest degree, the sensation which is connected with gaping. There can be no doubt that, if dyspnœa had arisen from any cause, the nostrils would have remained motionless, instead of assisting in that painful expression which is so often evident in asthmatic subjects.

After remaining four months in the hospital, the patient went out in the following state: The exostosis on the frontal bone had disappeared, the gonorrhœal discharge had ceased, and the general health was good. Her round and cheerful face expressed with vivacity every moral and physical sensation. Her laugh was still rather cold, for the motions of the lips did not appear to correspond with the rapidity or extent of the motions of the diaphragm and ribs. She masticated easily, and had the power of collecting her food into a mass for deglutition. With a slight effort she could completely close the eyelids, but the tears frequently flowed over the cheeks. The seton was kept in, and there was every reason to believe that in a few months the patient would retain only the remembrance of this singular affection.

If the experiments of Charles Bell upon the functions of the encephalic nerves required any confirmation from clinical observation, this case would be better calculated than any other to prove the correctness of his views respecting the use of the facial nerve. In this instance, all those phenomena occurred which result in animals from the division of that nerve at its exit from the styloid foramen. It is probable that in this patient a slight exostosis had compressed the nerves, as they passed out of the cranium. The efficacy of the antisyphilitic treatment is by no means certain. It is probable that the topical irritants performed the cure, and they were indispensable; for, very frequently after the removal of the cause which originally produced paralysis, this symptom still requires to be combated by local stimulants.

One part of the detail of this very curious case appears contradictory. At the commencement it is stated that the face was "*raide et insensible,*" while further on we are told that the power of motion only was affected, and that "*la sensibilité de la peau n'a éprouvé aucun changement.*" We presume that the term "*insensible,*" as first employed, was meant to describe an inanimate expression of the countenance, and not that the face was deprived of its physical sensation.—Editors.

